# Use of Endoscopic Ultrasound with Bronchoscope-Guided Fine-Needle Aspiration (EUS-B-FNA) in Pediatric Cases

**DOI:** 10.3390/children12050618

**Published:** 2025-05-09

**Authors:** Xiaowei Chen, Xiaofen Tao, Shuxian Li, Hujun Wu, Fang Jin, Lei Wu

**Affiliations:** 1 Department of Otorhinolaryngology-Head and Neck Surgery, The Children’s Hospital, Zhejiang University School of Medicine, National Clinical Research Center for Child Health, Hangzhou 310052, China; chenxiaowei@zju.edu.cn; 2 Department of Endoscopy Center, The Children’s Hospital, Zhejiang University School of Medicine, National Clinical Research Center for Child Health, Hangzhou 310052, China; icyshiny@zju.edu.cn (X.T.); doctor_shuxianli@zju.edu.cn (S.L.); 6515115@zju.edu.cn (H.W.); 21218423@zju.edu.cn (F.J.)

**Keywords:** pediatrics, mediastinal diseases, diagnostic technology

## Abstract

The intricate airway anatomy and poorly understood etiology of pediatric conditions render the diagnosis of mediastinal disorders particularly challenging. Traditional diagnostic methods, such as mediastinoscopy and thoracotomy, carry high risks and are often impractical in children. Endoscopic ultrasound with bronchoscope-guided fine-needle aspiration (EUS-B-FNA) enables a minimally invasive, esophageal route to mediastinal lesions, providing a diagnostic approach that is new to pediatrics compared with conventional techniques. We review the technical characteristics, clinical benefits, and prospective role of EUS-B-FNA in pediatric diagnostic investigation. We also review the clinical applications of EUS-B-FNA to date and aim to promote its use as a novel adjunct for the evaluation of complex mediastinal pathology in pediatric medicine.

## 1. Introduction

Infectious processes, neoplastic growths, and inflammatory conditions represent the predominant etiologies of mediastinal pathologies in pediatric populations. Accurate identification of the relevant diagnostic modalities is important for improving therapeutic strategies and preventive measures for mediastinal pathology because of the diverse etiological spectrum. Classic endoscopic techniques, such as mediastinoscopy, video-assisted thoracoscopic surgery (VATS), and thoracotomy, are related to considerable tissue damage and have difficult airway management challenges in children. Therefore, because of the intrinsic disadvantages of clinical and radiological evaluations, the establishment of safer and more effective diagnostic options for clinical use is essential [1].

Endobronchial ultrasound-guided transbronchial needle aspiration (EBUS-TBNA) is a minimally invasive method that has been widely used for diagnosing mediastinal disease in adult patients. However, this technique is not suitable for pediatric patients because of notable anatomical differences from adults, such as a narrower tracheal diameter. Moreover, the outer diameter of currently available EBUS bronchoscopes (typically 6.9–7.4 mm) exceeds the airway size in most children, particularly in infants and young children, making their use technically challenging and potentially hazardous. Endoscopic ultrasound with bronchoscope-guided fine-needle aspiration (EUS-B-FNA) appears to be becoming the preferred diagnostic choice because of the unique advantages that it has for diagnostic use in the pediatric population. This technique combines advanced imaging capabilities with direct and accurate access to the lesions through a fine-needle puncture, without entering the airway.

In this review, we systematically analyze the technical characteristics of EUS-B-FNA in the field of pediatrics and discuss its development and requirements.

## 2. History of EUS-B-FNA

Cytopathological diagnostic techniques have been continuously developed. Transbronchial needle aspiration (TBNA) is one of the earliest minimally invasive diagnostic techniques [2]. However, TBNA has an obvious disadvantage in that it cannot image in real time, resulting in poor sampling accuracy [3]. This defect requires doctors to be skilled. Therefore, the clinical application of TBNA is greatly limited. An important advance in diagnostic technology is the implementation of real-time visualization.

EBUS-TBNA greatly improves the success rate of the operation through real-time guidance of ultrasound [4]. This technique works well in adult diagnostics. However, in small children, the standard TBNA bronchoscope is not suitable for routine use because of their particular airway structure [5], and it may cause damage. The emergence of EUS-B-FNA has solved these technical difficulties [6]. The clinical use of EUS-B-FNA has proved that this technology has a good diagnostic effect for adults and children [6,7]. Notably, the refined application of this technique has enabled the successful diagnosis of diffuse large B-cell lymphoma in infant cases [8].

## 3. Potential Conditions Diagnosed by EUS-B-FNA

Building upon the clinical applications of EBUS-TBNA, EUS-B-FNA has shown considerable utility across diverse clinical scenarios, particularly in pediatric cases presenting with mediastinal masses or lymphadenopathy. The principal clinical indications for EUS-B-FNA in the pediatric population include the following.

### 3.1. Differential Diagnosis of Mediastinal Masses and Enlarged Lymph Nodes

EUS-B-FNA shows reliable efficacy in distinguishing benign lesions (e.g., cysts) from malignant neoplasms (e.g., lymphoma, neuroblastoma, and germ cell tumors), as shown by studies focusing specifically on pediatric cohorts [4,6,9].

This modality not only improves diagnostic precision but also yields critical information to guide therapeutic strategies. Lymphoma represents a frequent diagnostic consideration in pediatric patients presenting with mediastinal lymphadenopathy. However, because managing lymphoma requires precise histological subtyping, the adequacy of EUS-B-FNA specimens for comprehensive subclassification remains debatable [10].

### 3.2. Diagnosis of Pediatric Mediastinal Infectious Diseases

EUS-B-FNA has proven particularly useful in the diagnostic evaluation of complicated mediastinal infections in pediatric patients [9]. Following the acquisition of specimens, microbiological cultures and antibiotic sensitivity testing are routinely performed to establish the infectious etiology [11]. These findings subsequently guide the formulation of targeted therapeutic regimens.

### 3.3. Application in Tuberculosis Infections

In pediatric tuberculosis, mediastinal lymphadenopathy represents a common manifestation that can be accurately evaluated by EUS-B-FNA-guided specimen acquisition from affected lymph nodes. This minimally invasive approach yields high-fidelity specimens that are crucial for a definitive tuberculosis diagnosis. Post-diagnosis, drug susceptibility testing facilitates disease stratification [6], thereby permitting personalized therapeutic regimens and optimized clinical outcomes. This examination method has high reliability, which not only greatly improves diagnostic accuracy but also ensures a rapid and good therapeutic effect [12].

### 3.4. Other Applications 

EUS-B-FNA has a high image resolution and is useful for the diagnosis and treatment of cysts in children [9]. This technique can be used to localize and sample cysts accurately and quickly to determine the type of cyst formation, such as infection, congenital, or neoplastic. In addition, while traditional surgery is potentially dangerous for children, EUS-B-FNA is a safer, minimally invasive treatment method that can kill cysts effectively and avoid major surgery, and its treatment effect is better.

## 4. Comparison of Pediatric EUS-B-FNA with Other Techniques

### 4.1. Mediastinoscopy

Mediastinoscopy is a routine procedure for sampling mediastinal lesions, and this procedure can obtain large tissue samples and is useful for the diagnosis of complex diseases. Omer et al. believe that pediatric mediastinoscopy is an effective method of diagnosis because of the high rate of diagnosis and low rate of complications [13]. However, this procedure involves general anesthesia and has risks, such as bleeding, infection, pneumothorax, and other complications. In contrast, EUS-B-FNA is associated with less trauma, rapid recovery, good patient acceptance, and fewer complications than mediastinoscopy. However, the negative predictive value of EUS-B-FNA is lower than that of surgical sampling, and additional methods (e.g., mediastinoscopy or surgical biopsy) may be required when the sample result is negative to improve diagnostic accuracy [5].

### 4.2. VATS

VATS is now increasingly used in pediatric patients [14]. This technique is used to diagnose mediastinal disease and to help treat it. VATS provides enhanced visualization capabilities, enables the acquisition of larger tissue specimens, and permits complete lesion resection when malignancy is suspected compared with EUS-B-FNA [15]. Although EUS-B-FNA is primarily restricted to posterior mediastinal and paratracheal biopsies, it is associated with reduced tissue trauma and facilitates quicker postoperative recovery. Notably, pediatric VATS typically requires one-lung ventilation, with airway management difficulties escalating inversely with the patient’s age [14]. Therefore, EUS-B-FNA is a safer alternative for younger pediatric patients. The selection between these two techniques should be based on careful consideration of the patient’s age, anatomical lesion characteristics, and potential therapeutic requirements.

### 4.3. Percutaneous Ultrasound-Guided Mediastinal Puncture

Conventional ultrasound imaging shows diagnostic utility primarily for pleural effusions and large chest wall-adherent masses. Bhalla et al. showed that percutaneous mediastinal ultrasound can identify lymph nodes at specific nodal stations, but it fails to visualize those at stations 8, 9, and 11–14 [16]. When these lymph nodes are obscured by intervening lung parenchyma, vascular structures, or cardiac shadowing, percutaneous ultrasound-guided biopsy becomes technically unfeasible. In contrast, EUS-B-FNA provides superior visualization of lymph nodes at stations 8 and 9 because of their esophageal proximity.

### 4.4. EBUS-TBNA

EUS-B-FNA and EBUS-TBNA are minimally invasive, ultrasound-guided endoscopic techniques designed for mediastinal lymph node sampling. EBUS-TBNA is primarily indicated for lymph node biopsy in the tracheal and carinal regions, such as stations 2, 4, 7, 10, 11, and 12. In contrast, EUS-B-FNA shows superior accessibility for left-sided, posterior, and inferior mediastinal lymph nodes, particularly stations 8 and 9. The complementary use of both techniques optimizes the diagnostic yield for mediastinal lymph node evaluation. However, pediatric pulmonologists generally show greater familiarity and technical comfort with the airway-based EBUS-TBNA than with EUS-B-FNA. However, EUS-B-FNA remains essential for younger pediatric patients, hypoxic individuals, patients with EBUS-TBNA-inaccessible lymph nodes, and patients contraindicated for bronchoscopy due to respiratory compromise [17]. Consequently, proficiency in both techniques is essential for pediatric pulmonologists. The development of a compact (5.9 mm) EBUS scope prototype is expected to considerably facilitate EBUS-TBNA/EUS-B-FNA procedures in younger pediatric populations [17,18].

## 5. EUS-B-FNA Procedure

### 5.1. Preparation of Equipment

#### 5.1.1. EBUS Bronchoscopes

Currently, EBUS bronchoscopes are manufactured by three leading companies, namely Olympus (Tokyo, Japan), Pentax (Tokyo, Japan), and Fujifilm (Tokyo, Japan) [10]. Multiple models of flexible endoscopes with distinct characteristics are commercially available (Table 1).

#### 5.1.2. Needles

EBUS-TBNA is performed using the following four needle sizes: 19-, 21-, 22-, and 25-gauge. All needles incorporate a detachable stylet with a sharp or smooth retractable tip, housed within a protective sheath and featuring a distal suction port. The 19-gauge needle provides superior tissue yield, despite its reduced flexibility. In contrast, the 25-gauge needle offers enhanced maneuverability and puncture precision. Notably, the needle gauge (21- vs. 22-gauge) does not affect the diagnostic yield in TBNA procedures. Larger-gauge needles enable the acquisition of more substantial specimens suitable for ancillary testing, such as immunohistochemistry and molecular analysis [10].

### 5.2. Procedural Steps

**Anesthesia**: In pediatric populations, achieving sufficient anesthesia is imperative to ensure the well-being of the child and the efficacy of the procedure. Keeping children comfortable under appropriate sedation with continuous ventilation throughout the sampling procedure is necessary because of the narrow tolerance for error during needle insertion [19]. Depending on the patient’s condition, several anesthesia methods can be selected as discussed below.

**Deep Sedation**: This technique obviates the need for endotracheal intubation, providing a more streamlined procedure and better visibility of the upper airway, which facilitates the insertion of the EBUS bronchoscope more easily. Consequently, deep sedation is frequently used in clinical practice.

**Endotracheal Intubation Anesthesia**: Endotracheal intubation improves pulmonary management. However, endotracheal intubation anesthesia potentially reduces the accessibility of the upper larynx compared with deep sedation. This reduced accessibility, in turn, increases the difficulty of inserting an EBUS bronchoscope into the esophagus.

Although laryngeal mask anesthesia is the preferred choice for EBUS-TBNA procedures, it is not suitable for EUS-B-FNA because the laryngeal mask interferes with access to the esophageal opening.

**Figure 1:**
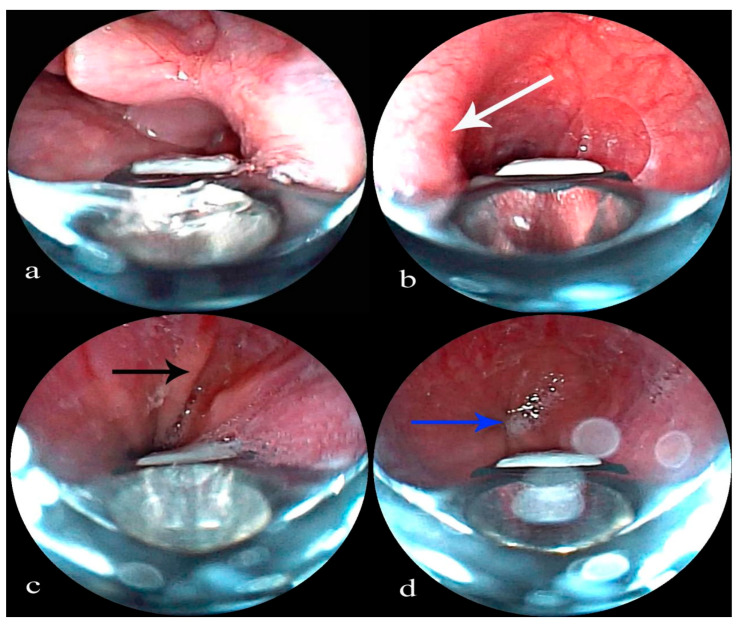
(**a**) An EBUS bronchoscope is advanced below the glottis into the esophagus. (**b**) Aortic arch impression (white arrow). (**c**) The Z-line (black arrow). (**d**) Gastric cardia (blue arrow).

**Insertion and Esophageal Approach**: Oxygen is administered via a nasal cannula, and the patient is carefully positioned in a slightly lateral decubitus position [6]. An EBUS bronchoscope is skillfully inserted orally, passed beneath the glottis, and then directed into the esophagus (Figure 1a). The unique anatomical features visible during esophageal endoscopy serve as reliable markers for the bronchoscope’s location. These markers include the following: a rhythmic protrusion on the lateral aspect of the middle esophagus, corresponding to aortic pulsation, which indicates an aortic arch impression (Figure 1b); the Z-line, which is clearly defined at the junction between the lower esophagus and the gastric cardia (Figure 1c,d); and a constriction at the site where the esophagus passes through the diaphragmatic hiatus.

## 6. Lesion Sampling

### 6.1. Activation of the Transducer and Placing It Adjacent to the Target Lesion

The up-down lever is adjusted to minimize the distance from the esophageal wall. Ultrasound and color Doppler are used to localize the lesion, evaluate the presence of blood vessels, and characterize its size, echogenicity, and structure. A puncture guide is used to design a safe sampling trajectory, avoiding blood vessels.

### 6.2. Inserting the Needle and Catheter Assembly

**Figure 2:**
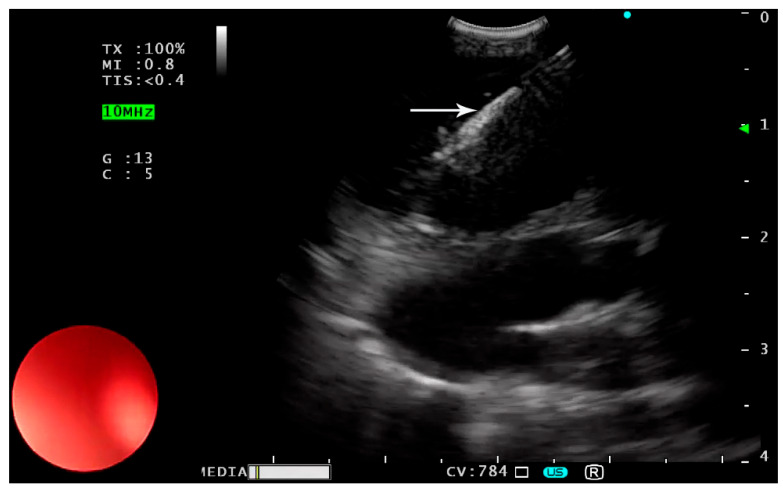
Insertion of the puncture needle (white arrow) into the target mass.

The sheath is advanced until it is visible on the screen. The puncture depth is adjusted and locked. Subsequently, the needle is precisely directed into the target under ultrasound visualization (Figure 2).

### 6.3. Sample Aspiration

After withdrawing the puncture needle’s stylet, a syringe is used to apply negative pressure while gently oscillating the needle within the lesion site. If blood appears in the syringe during this process, the procedure should be immediately stopped. Once sampling is completed, the vacuum is released and the needle is retracted and placed securely in the sheath. The puncture depth is adjusted and locked, and the needle is raised and removed from the operating channel. Typically, three passes are carried out for sufficient sampling. According to the EBUS-TBNA protocol, if the tissue obtained is still insufficient, a maximum of five attempts can be made [3].

### 6.4. Sample Preparation

The specimen is divided into two parts. One part is used for slide preparation, which is fixed in 95% ethanol for cytology and stained for on-site assessment. The other part is used for preparing a cell block, which allows for further pathological, immunohistochemical, or microbiological tests. A 3–5 mL sterile saline rinse is used to preserve the fluid for microbiological evaluation.

### 6.5. Post-Procedure Care

A radiological examination is not required unless esophageal perforation is suspected (e.g., mediastinal emphysema) or the patient shows symptoms such as chest pain, dyspnea, or fever.

Strict aseptic techniques and continuous real-time ultrasound monitoring are fundamental to ensuring the patient’s safety. In complex cases involving deep or vascular-adjacent lesions, comprehensive risk assessment and contingency planning are important.

## 7. Contraindications

In addition to the general contraindications for bronchoscopy, the primary contraindications for EUS-B-FNA encompass patients who are unable to tolerate moderate sedation or general anesthesia and those determined to be at high risk according to the Pediatric Bleeding Questionnaire assessment. Patients with structural esophageal abnormalities (e.g., strictures or a high risk of perforation) should not use this technique. The reason for this recommendation is that the EBUS bronchoscope is not able to sufficiently dilate the esophagus, which could lead to limited visibility during the procedure [4]. Esophageal varices are also a contraindication [20]. Additionally, hemodynamically unstable patients need to be excluded from this particular procedure because it might worsen their condition unless the diagnosis is urgent and there is no other way to access the information.

## 8. Complications

EUS-B-FNA is a minimally invasive method, but there can be complications. Adverse effects (e.g., bleeding from the site of puncture and infectious complications) can occur. Cystic lesions have a risk of infection and bacteremia. To avoid these complications, antibiotic prophylaxis should be applied [21]. Esophageal perforation is a potential complication, with a risk of 0.02% in adult EUS cases [22]. However, there have been no reported cases of esophageal perforation associated with pediatric EUS-B-FNA. Theoretically, an esophageal tear may also occur during aspiration of the node while the EBUS needle traverses the esophageal wall. To avoid this complication, the child should be deeply sedated [5].

Although rare, extensive preoperative assessment, sterile intervention, and preventive interventions are crucial in high-risk individuals to decrease the risk of these complications.

## 9. Limitations and Potential Concerns

Although EUS-B-FNA has advantages in the diagnosis of mediastinal diseases in children, its limitations and potential problems still need to be considered. This technique may increase the risk of esophageal injury and bleeding in children with a thin esophageal wall or anatomical abnormalities in the vascular structure [4,22].

One main limitation of EUS-B-FNA is its limited sample size. Mediastinoscopy and surgical thoracoscopy can remove larger specimens than EUS-B-FNA. In diseases such as lymphoma [10], there might not be sufficient tissue samples to perform immunohistochemical staining or genetic profiling. In lymphoma with a high suspicion, if EUS-B-FNA sampling does not reach the threshold of appropriate specimens, mediastinoscopy or surgical biopsy should be conducted for optimal diagnostic accuracy.

Moreover, the complication rate of EUS-B-FNA is comparatively low, but there is still a risk of infection after the operation and local hemorrhage or esophageal perforation during operation, which should be carefully handled [21,22]. The patient’s coagulation function should be assessed before surgery to determine whether the esophageal structure is abnormal (e.g., esophageal strictures or varices), especially to reduce the complications during surgery [20]. From a technical point of view, EUS-B-FNA is not easy to learn and has to be performed by specially trained doctors. The outcome and accuracy of diagnosis will be affected if the operation is not performed well. Therefore, future research and development should focus on optimizing special equipment suitable for children, improving the operation technology, and promoting multi-disciplinary cooperation, so that this technology can be used more frequently in clinical practice.

## 10. Technical Challenges and Future Directions

Mediastinal diseases in children are complex and require the cooperation of doctors from multiple departments to diagnose and treat them. Only when doctors of various departments cooperate can they make a more accurate diagnosis and formulate a better treatment plan.

EUS-B-FNA is an emerging technology. This technology will take a long time to be widely used. If experts in children’s bronchoscopy want to learn further, they must first master EUS-B-FNA technology under the guidance of senior experts. In addition, continuing to consult and learn from experienced doctors is important [1].

Sharma et al. successfully examined a neonate’s stomach using EUS-B-FNA technology. This finding proves that the technology is versatile and practical [23]. In addition to mediastinal masses and periesophageal lymph nodes, EUS-B-FNA can also be used to safely obtain samples of pulmonary nodules near the esophagus [18,24] and pericardial effusion [25]. EUS-B-FNA has great application potential in the field of pediatrics and is expected to be greatly expanded. However, much more research is required to evaluate the application of this technology in the health assessment of young patients.

## 11. Conclusions

EUS-B-FNA is a minimally invasive technique with good safety and diagnostic capability and is thus widely recognized. EUS-B-FNA plays a key role in assessing mediastinal lymphadenopathy in children. This technique should be the first choice for evaluating mediastinal lesions in children. Mediastinoscopy or VATS should be considered only when EBUS-TBNA or EUS-B-FNA cannot yield a diagnosis, or when the lesion is inaccessible by either method.

Although EUS-B-FNA has encountered some technical difficulties in the application of pediatric medicine, its prospects are still good. With the continuous improvement of equipment and operation technology, the potential for large-scale application of this technology will be further enhanced.

## Figures and Tables

**Table 1:** Equipment for endobronchial ultrasound.

Endoscope	Scope Length (mm)	Outer Diameter (mm)	Outer Diameter of Tip (mm)	Working Channel (mm)	Electronic Probe Scanning	Bending Angle (Up/Down)	Field of View Direction	Imaging Mode
BF-UC290F (Olympus, Tokyo, Japan)	600	6.3	6.6	2.2	65°convex	160°/70°	20°	Composite
EB19-J10U (Pentax, Tokyo, Japan)	600	6.3	7.3	2.0	75°convex	120°/90°	45°	Front-View CCD
EB-530US (Fujifilm, Tokyo, Japan)	610	6.3	6.7	2.0	65°convex	130°/90°	10°	Front-View CCD
